# Chitosan and its derivatives as promising plant protection tools

**DOI:** 10.18699/VJGB-23-116

**Published:** 2023-12

**Authors:** A.B. Shcherban

**Affiliations:** Kurchatov Genomic Center of ICG SB RAS, Novosibirsk, Russia

**Keywords:** plant protection products, pesticide, chitosan, novohizol, pathogen, resistance, yield, средства защиты растений, пестицид, хитозан, новохизоль, патоген, устойчивость, урожайность

## Abstract

In modern conditions, the increase in the yield of agricultural crops is provided not by expanding the areas of their cultivation, but mainly by introducing advanced technologies. The most effective strategy for this purpose is the development of genetically resistant and productive cultivars in combination with the use of a variety of plant protection products (PPPs). However, traditional, chemical PPPs, despite their effectiveness, have significant drawbacks, namely, pollution of environment, ecological damage, toxicity to humans. Recently, biological PPPs based on natural compounds have attracted more attention, since they do not have these disadvantages, but at the same time they can be no less effective. One of such agents is chitosan, a deacetylation product of chitin, one of the most common polysaccharides in nature. The high biological activity, biocompatibility, and safety of chitosan determine the breadth and effectiveness of its use in medicine, industry, and agrobiology. The review considers various mechanisms of action of chitosan as a biopesticide, including both a direct inhibitory effect on pathogens and the induction of plant internal defense systems as a result of chitosan binding to cell surface receptors. The effect of chitosan on the formation of resistance to the main classes of pathogens: fungi, bacteria, and viruses has been shown on a variety of plant objects. The review also discusses various ways of using chitosan: for the treatment of seeds, leaves, fruits, soil, as well as its specific biological effects corresponding to these ways. A separate chapter is devoted to protection products based on chitosan, obtained by its chemical modifications, or by means of combining of a certain molecular forms of chitosan with various substances that enhance its antipathogenic effect. The data presented in the review generally give an idea of chitosan and its derivatives as very effective and promising plant protection products and biostimulants.

## Abstract (ru)

В современных условиях прирост урожайности сельскохозяйственных культур обеспечивается не за счет расширения площадей их возделывания, а главным образом благодаря внедрению передовых технологий. Наиболее эффективная стратегия включает создание генетически устойчивых к неблагоприятным факторам и продуктивных сортов в сочетании с использованием разнообразных средств защиты растений. Однако традици- онные, химические, средства защиты, несмотря на эффективность, имеют существенные недостатки: загрязнение окружающей среды, нарушение экологии, токсичность для человека. В последнее время все больше внимания привлекают биологические (на основе природных соединений) средства защиты растений – они лишены этих не- достатков, при этом могут быть не менее эффективными. К таким средствам относится хитозан – продукт деаце- тилирования хитина, одного из наиболее распространенных в природе полисахаридов. Высокая биологическая активность, биосовместимость и безопасность хитозана определяют широту и эффективность его применения в медицине, промышленности и агробиологии. В обзоре рассмотрены механизмы действия хитозана в качестве биопестицида, включающие как прямое подавляющее воздействие на патогены, так и индукцию внутренних за- щитных систем растения в результате связывания хитозана поверхностными рецепторами клеток. На множестве растительных объектов показано влияние хитозана на формирование устойчивости к основным классам пато- генов: грибам, бактериям и вирусам. Кроме того, в работе оценены способы применения хитозана, включающие обработку семян, листьев, плодов, почвы, а также соответствующие этим методам специфические биологические эффекты. Отдельный раздел посвящен средствам защиты на основе хитозана, полученным как путем его химиче- ской модификации, так и с помощью комбинирования тех или иных молекулярных форм с различными вещества- ми, усиливающими его антипатогенное действие. Представленные в обзоре данные дают представление о хито- зане и его производных как об эффективных и перспективных средствах защиты растений и биостимуляторах.

## Introduction

The intensive growth of the world’s population poses a global problem for agriculture to increase the yield of the main cultivated plant crops. However, yield losses due to numerous bio- and abiotic factors can be very significant. Particularly actual is the control of various pathogens: bacteria, viruses, fungi, which not only reduce yields, but also reduce the quality of plant products as a result of the accumulation of toxins and other metabolites during the infectious process. For a long time this control has been carried out through the use of chemical pesticides, which cover a wide range of pests, are easy to use and have a low cost. But, along with this, they greatly pollute the environment and negatively affect human health (Igbedioh, 1991). In addition, their accumulation in the environment and living organisms can lead to irreversible consequences in ecosystems and a decrease in biodiversity (Yasmin, D’Souza, 2010). The effect of chemical plant protection products can be significantly weakened due to the emergence of resistant forms of pathogens, which makes it necessary to increase the rate of use of these agents or to create new ones (Kumaraswamy et al., 2018).

Another direction is the creation of new plant varieties that are genetically resistant to stress factors and have increased yields in various environmental conditions. However, although this method is the most reliable and effective means of protection, it can also have a temporary effect due to the emergence of new aggressive forms of pathogens. A typical example is the emergence of a new Uganda 99 race of stem rust, a dangerous fungal pathogen of cereals (Singh et al., 2011). In addition, there is a risk of transfer from other areas of such forms of pests to which certain varieties are susceptible

Apparently, the most effective strategy for plant protection is a combination of methods for the formation of genetic resistance with the use of biostimulants, or biopesticides, which, unlike chemical pesticides, do not cause environmental pollution, ecosystem changes and a negative impact on human health, but are no less effective (Tyuterev, 2014). Over the past decades, a number of biostimulants have been developed that are used to control the processes of plant growth and development, increase their productivity, and also reduce sensitivity to pathogens (Rouphael, Colla, 2020). Among them, a special place is occupied by chitosan, a product of the processing of chitin, the second most widespread natural biopolymer after cellulose.

The aim of this review is to analyze the accumulated scientific data on the effectiveness of the use of chitosan and its derivatives to control plant diseases and increase their productivity. The mechanisms of induction of plant resistance to stress factors under the influence of these plant protection agents are discussed.

## Chitosan

**Fig. 1.**
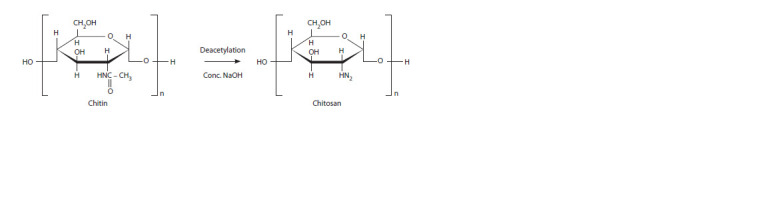
The structure of chitin and its derivative – chitosan

The precursor of chitosan is chitin, a biopolymer of the group of nitrogen-containing polysaccharides, consisting of N-acetyl-D-glucosamine and D-glucosamine (Fig. 1). Chitin forms the external skeleton of most invertebrates and is also a component of the cell walls of fungi, yeasts, and algae, accounting for up to 16% of the body’s dry weight as a structural polysaccharide (Muzzarelli, 2010).

The use of chitosan began in the 80s of the last century, and since then there have been many works devoted to its use in chemistry, medicine, and agrobiology (Rinaudo, 2006; Malerba, Cerana, 2016). These applications are due to the unique physicochemical properties of chitosan, such as: biocompatibility, non-toxicity and biodegradation. Some organisms, such as zygomycetes, are capable of synthesizing chitosan in significant amounts, which allows to use them to obtain this valuable chitin derivative in various fields of biotechnology (Karimi, Zamani, 2013).

In industry, chitosan is usually obtained from chitin by deacetylation during a chemical process using NaOH (Skryabin et al., 2002). The products of this process are very heterogeneous in terms of the degree of deacetylation, molecular weight, and other chemical parameters determining the differences in their physical properties (viscosity, solubility), which, in turn, determine the possibilities of using chitosan and its biological effects (Orzali et al., 2017). In medicine, it is successfully used for tissue regeneration due to its ability to form elastic biofilms on the wound surface; it has also found application in the creation of anticoagulant and antisclerotic drugs (Skryabin et al., 2002; Chen et al., 2021). Among other applications there are cosmetics, food processing, wastewater treatment, environmental protection (Morin-Crini et al., 2019). In many countries, chitosan and its derivatives have been used for a long time as biostimulants that increase plant productivity and their resistance to pathogens (Tyuterev, 2015). All these effects of chitosan, along with its availability and relatively low cost, make its use as a biological plant protection product economically viable and justified (Xing et al., 2015).

## Chitosan as an inducer of plant immunity

The induction of the internal mechanism of plant protection against pathogens is an effective and safe alternative to chemical methods of protection. It is known that a number of substances can enhance resistance to pathogens as elicitors (Gaffney et al., 1993; Malerba, Cerana, 2016). The polysaccharide chitosan is one of the most effective resistance stimulators (Falcón-Rodríguez et al., 2012). Its mechanism of action is not yet well understood. It is assumed that chitosan binds to transmembrane cell receptors, which are not currently identified. Also, no protein kinase cascades transmitting a signal from receptors to transcription factors or protection genes have been identified. Various models have been proposed to explain the role of chitosan in plant immunity (Orzali et al., 2017). The most common model suggests the induction of nonspecific PAMP (pathogen-associated molecular pattern) by chitosan, an immune system that includes a number of interrelated signaling cascades (Tyuterev, 2002; Tang et al., 2012). The central role in this system is played by hormonal pathways associated with the synthesis of salicylic and jasmonic acids (SA and JA). In particular, the octadecanoid pathway is activated, leading to the accumulation of JA in tissues (Ishiguro et al., 2001). This hormone, along with SA, activates defense genes encoding various PR (pathogenesis related) proteins (Reinbothe et al., 2009). 

Another pathway is initiated by the accumulation of free oxygen radicals (ROS, reactive oxygen species), which are formed in tissues at the earliest stage of stress. Besides the direct toxic effects on pathogens, ROS are functioning as cell signaling molecules that trigger plant defense responses such as cell wall strengthening, hormone synthesis, and programmed cell death (Grant, Loake, 2000). The development of systemic resistance also involves the nitric oxide (NO) signaling pathway, which activates an early protective response, including a hypersensitivity reaction, the formation of a callose layer and the expression of a number of proteins: PR-1 and PR-5, chitinase (CHI), polyphenol oxidase (PPO), peroxidase (POX), superoxide dismutase (SOD), catalase (CAT), and phenylalanine ammonium lyase (PAL) (Manjunatha et al., 2008, 2009). Enzymes PPO, POX, SOD, and CAT are the main enzymes that neutralize excess oxygen radicals (Elsharkawy et al., 2022). PAL is involved in the biosynthesis of protective phenolic compounds such as flavonoids, phenylpropanoids, and lignin (Appert et al., 1994).

**Fig. 2.**
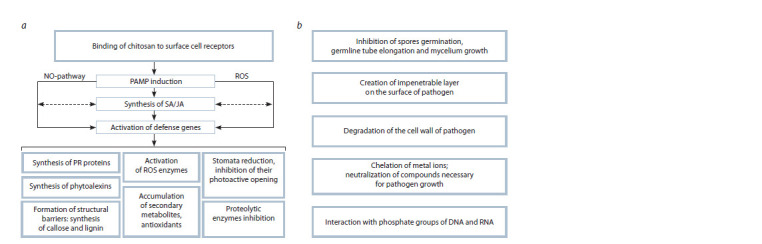
The effect of chitosan on plant defense mechanisms (a) and its antipathogenic effects (b).

As a result of treatment with chitosan, phytoalexins, low molecular weight antibiotic substances, accumulate in plant tissues (Hadwiger, 2013). The synthesis of callose, a polysaccharide, is also induced, which is deposited in the cell wall and serves as a barrier to the penetration of pathogenic organisms (Köhle et al., 1985; Conrath et al., 1989). The process of lignification, which is enhanced under the influence of chitosan, serves the same purpose (Hirano et al., 1999). In particular, it was shown that the formation of structural barriers to the path of the pathogen is the main plant response to chitosan in the tomato Solanum lycopersicum L. (Benhamou et al., 2001). Under the influence of chitosan, the suppression of proteolytic enzymes released by pathogens for penetration into plant tissues is enhanced (Peña-Cortes et al., 1988). The effect of chitosan also manifests itself in the reduction of the size of stomata as a result of a decrease in their sensitivity to light (Lee et al., 1999). Possibly, this effect is related to the hormonal activity of JA similar to that of abscisic acid, which is a key regulator of the transpiration process (Sembdner, Parthier, 1993). Other authors have revealed the role of chitosan in the biosynthesis of curcumin, a powerful natural antioxidant deposited in the root tissue of turmeric Curcuma longa L. (Sathiyabama et al., 2016). Thus, a wide range of regulatory effects was established that enhance plant immunity under the treatment with chitosan (Fig. 2, a).

In addition to the eliciting effect on plant cells, chitosan is able to have a direct effect on pathogens.

## Mechanisms of antipathogenic action of chitosan

Chitosan exhibits a variety of antipathogenic activity, which depends, on the one hand, on its chemical properties and method of preparation, and, on the other hand, on the characteristics of the host plant and environmental conditions. In some studies, oligomeric forms of chitosan (penta- or heptamers) exhibited higher fungicidal activity compared to larger molecules (Rabea et al., 2003), while in other studies, on the contrary, an increase in the antipathogenic effect was observed with increasing molecular weight (Kulikov et al., 2006).

Unlike natural chitin, the molecules of which are not charged and have no antimicrobial activity, chitosan has a positive charge. According to one model, electrostatic interaction of chitosan molecules with negatively charged surfaces of pathogen cells results in an increase in the permeability of plasma membranes and destruction of the cell wall (Je, Kim, 2006). Another mechanism implies the formation of an impermeable chitosan polymer layer on the cell surface, which prevents the absorption of nutrients and, at the same time, the excretion of metabolites into the intercellular space (Xing et al., 2015). Chitosan is also able to chelate metal ions and some nutrients necessary for the development of bacteria or fungi, thereby inhibiting the reproduction of the latter and the production of toxins by them (El Hadrami et al., 2010; Xing et al., 2015). In a number of works, the inhibitory effect of chitosan on various stages of pathogen development was established (Rabea et al., 2005; Meng et al., 2010; Reglinski et al., 2010; Badawy, Rabea, 2011). The mechanisms of the antipathogenic action of chitosan are shown in Fig. 2, b.

## The use of chitosan for protection against various pathogens

Due to climate change, over the past 10–15 years, there has been an increasingly intensive development of various infectious diseases of the main crops of plants, which has led to a significant drop in their productivity and a decrease in product quality. The most widespread are fungal diseases, which account for more than 80 % of all diseases of agricultural plants (Garibova, Sidorova, 1997). So, for example, common wheat Triticum aestivum L. (2n = 42) can be affected by 25 fungal diseases, including smut, rust, root rots, etc. Yield losses from these diseases in separate areas of distribution can reach 70% or more (Singh et al., 2011).

Under in vitro conditions, the fungicidal effect of chitosan was shown against a number of pathogenic fungi, representatives of the genera Botrytis, Alternaria, Colletotrichum, Rhizoctonia, etc. (Orzali et al., 2017). At the same time, the suppressive effect of chitosan on various stages of fungal development was demonstrated: mycelium growth, sporulation stage, viability of spores and the efficiency of their germination, and the ability of fungus to produce virulence factors (Badawy, Rabea, 2011). For example, chitosan completely inhibited spore germination and mycelial growth in Alternaria kikuchiana S. Tanaka and Physalospora piricola Nose (Meng et al., 2010). Also, in grape, it effectively suppressed the growth of mycelium of the fungus Botrytis cinerea Pers in vitro, as well as on leaves and fruit clusters (Reglinski et al., 2010). E.I. Rabea et al. (2005) reported increased fungicidal activity of 24 chemically modified chitosan derivatives compared to conventional chitosan in a radial growth model of hyphae of B. cinerea and Pyricularia grisea fungi. Other authors showed that chitosan is able to penetrate the plasma membrane of Neurospora crassa Shear and cause cell death as a result of energy imbalance (Palma-Guerrero et al., 2009). An increase in the resistance of tomato to Alternaria under the influence of chitosan was demonstrated (Bayrambekov et al., 2012). Its effectiveness against the anthracnose pathogen (Colletotrichum sp.) in cucumbers is comparable to that of chemical fungicides (Dodgson J.L.A., Dodgson W., 2017). Chitosan treatment of common wheat plants prior to infection with the fungal pathogen Fusarium graminearum Schwabe, the causative agent of Fusarium rot, has been shown to significantly reduce the number of affected ears (Kheiri et al., 2016). In the same culture, the effect of chitosan on resistance to another dangerous fungal disease, brown leaf rust caused by Puccinia triticina Erikss., was shown (Elsharkawy et al., 2022).

Chitosan and its derivatives inhibit the growth of various bacteria (Fei Liu et al., 2001; Wiśniewska-Wrona et al., 2007; Rabea, Steurbaut, 2010; Badawy et al., 2014). However, the latter are less sensitive to the action of chitosan than fungi (Kong et al., 2010). Its minimum inhibitory concentration varies from 0.05 to 0.1 % depending on the type of bacteria, the molecular weight of chitosan, and the pH of the solution (Katiyar et al., 2014). Some authors showed a stronger effect of chitosan on Gram-positive bacteria compared to Gramnegative ones (No et al., 2002; Tayel et al., 2010). This can be explained by the fact that the latter form an additional outer membrane, which is impermeable to high molecular weight chitosan (Xing et al., 2015). However, as shown in other studies, under certain conditions (pH, Mg2+ content), chitosan is able to overcome this barrier, making Gram-negative bacteria more sensitive to its action (Helander et al., 2001). Chitosan negatively affects the growth of a number of pathogenic bacteria, including Xanthomonas (Li et al., 2008), Pseudomonas syringae van Hall (Mansilla et al., 2013), Agrobacterium tumefaciens (Smith et Townsend) Conn. and Erwinia carotovora (Jones) Waldee (Badawy et al., 2014). The antimicrobial activity of chitosan derivatives against Escherichia coli Migula and Staphylococcus aureu Rosenbach was also shown (Su et al., 2009).

There are a lot of works devoted to the antiviral effects of chitosan (Su et al., 2009). In plants, chitosan induces resistance to viral diseases, preventing the spread of viruses and viroids so that most treated plants do not develop a systemic viral infection (Chirkov, 2002). It was found that chitosan enhances the expression of RNases associated with the development of resistance to potato virus X (PVX), suppressing its replication in cells (Iriti, Varoni, 2015). Chitosan-treated tomato plants not only show resistance to tomato mosaic virus, but also increased vegetative growth (Abd El-Gawad, Bondok, 2015). Chitosan also effectively inhibits the development of alfalfa mosaic virus (AIMV), tobacco mosaic virus (TMV), squash mosaic virus (SMV) (Nagorskaya et al., 2014). The level of suppression of viral infection varies depending on the molecular weight of chitosan. Low molecular weight chitosan suppresses the formation of local necrosis caused by TMV in tobacco by 50–90 % (Davydova et al., 2011).

Examples of the protective action of chitosan against various plant pathogens are given in the Table. The defense reaction induced by chitosan depends not only on the type of plant or pathogen, but also on the conditions and method of its application

**Tab. 1.**
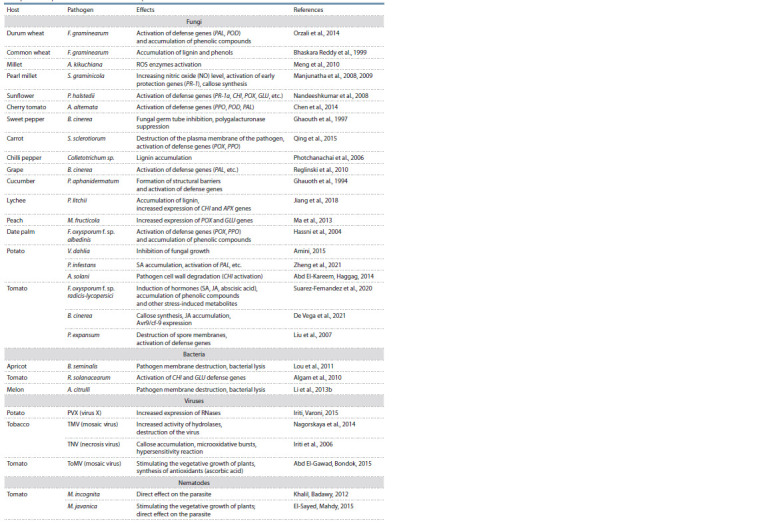
Examples of the protective action of chitosan in plants

## Methods of chitosan application

Seed treatment

There are many examples of the effect of seed treatment on plant resistance to infections (Benhamou et al., 1994; Algam et al., 2010; Amini, 2015). In most cases, low molecular weight chitosan demonstrated the highest efficiency (Orzali et al., 2017). Mechanisms for increasing resistance in this case differ depending on the pathogen. For example, it was shown that the treatment of pearl millet seeds with a 4 % solution of chitosan increased resistance to downy mildew caused by the oomycete Sclerospora graminicola (Sacc.) J. Schröt (Sharathchandra et al., 2004) by 48 %. In addition, an increase in the expression of a number of proteins associated with the NO signaling pathway was found (see above). A similar effect of seed treatment was found in sunflower in relation to the causative agent of downy mildew Plasmopara halstedii (Farl.) Berl. et de Toni (Nandeeshkumar et al., 2008). Chitosan treatment of T. aestivum seeds increased resistance to obligate phytopathogens due to the accumulation of phenolic compounds and lignification of cell walls at subsequent stages of plant development after germination (Bhaskara Reddy et al., 1999). An intensification of the lignification process was found during the treatment of chili pepper seeds with chitosan, which increased the survival rate of seedlings infected with the anthracnose pathogen (Photchanachai et al., 2006). Seed treatment with chitosan induced resistance in tetraploid wheat Triticum durum Desf. to the causative agent of Fusarium F. graminearum (Orzali et al., 2014). At the same time, the analysis of plant tissues showed an increase in the activity of enzymes: guaiacol-dependent peroxidase (POD), ascorbatedependent peroxidase (APX), as well as PPO and PAL.

Besides the antipathogenic effect, the effect of seed treatment with chitosan is based on the enhancement of metabolic processes in host plant. Thus, it was shown that soaking wheat seeds in a solution of chitosan (in the form of a poly- or oligomer) increased the length of the stem and roots in seedlings 6 days after treatment (Krivtsov et al., 1996). Later, these data were confirmed by Chinese authors, who found that treatment with low molecular weight chitosan increases the vigor of wheat seed germination, as well as plant viability, biomass, and yield, which is associated with accelerated carbon and nitrogen metabolism (Zhang et al., 2017).

Treatment of soil

It is assumed that the addition of chitosan improves soil structure, and also affects the ratio of soil microorganisms, shifting it towards beneficial ones. There is evidence of an increase in the population of actinomycetes and pseudomonads, as well as Bacillus subtilis in soils treated with chitosan (Mulawarman et al., 2001). The latter also favorably affects the growth of mycorrhizal fungi (Park, Chang, 2012). In addition, chitosan is able to chemically neutralize toxic substances, pesticides, and fertilizers (Xing et al., 2015). The positive effect of chitosan in the soil also includes the induction of plant defense mechanisms against soil pathogens. For example, in tomato, significant inhibition of the pathogenic fungus Fusarium oxysporum f. sp. radicis-lycopersici and nematode Meloidogyne javanica Treub was observed as a result of depolarization of root cell membranes that produce hormones, signal lipids, and various protective substances, including phenolic compounds (Suarez-Fernandez et al., 2020). However, in another work, it was shown that the treatment of roots with chitosan did not affect the development of fusariosis in sensitive celery varieties, but effectively reduced the manifestations of the disease in a tolerant variety (Bell et al., 1998).

Chitosan, applied as soil drainage, controlled the development of the bacterial pathogen Ralstonia solanacearum Smith in tomato, both as a result of direct action on the pathogen and through eliciting effects, such as the synthesis of CHI and B-2,3-glucanase (GLU), an enzyme that decomposes large polysaccharides (Algam et al., 2010). Soil treatment with chitosan effectively controlled the development of late blight in sweet pepper (Kim et al., 1997) and strawberries (Eikemo et al., 2003). In the date palm, chitosan activated such enzymes as POD and PPO in root cells, as well as the production of hydroxycinnamic acid, which promotes resistance to F. oxysporum f. sp albedinis (Hassni et al., 2004). There are a number of works showing the high efficiency of chitosan applied to the soil to control nematodes of various species, so that its action reduces the nematode population, egg weight, and the degree of root damage (Khalil, Badawy, 2012; El-Sayed, Mahdy, 2015).

Leaf treatment

Treatment of vegetative plants with chitosan has been used for many species for various purposes. For example, in the barley Hordeum vulgare L., it caused an oxidative burst and production of phenolic compounds in the leaves, which created an unfavorable environment for the spread of fungi (Faoro et al., 2008). Processes such as callose accumulation, microoxidative bursts, and hypersensitivity reaction also developed during tobacco leaf treatment, which ensured its resistance to tobacco necrosis virus (TNV) (Iriti et al., 2006). In another study, the effects of chitosan formulations on the suppression of powdery mildew in grapes were studied (Iriti, Varoni, 2015). In tomato, treatment of leaves with a solution of chitosan caused resistance to the pathogenic fungus B. cinerea (De Vega et al., 2021). This resistance correlated with callose deposition at sites of infection, JA accumulation, and expression of the Avr9/cf-9 elicitor protei

In cucumber leaves, chitosan activated a number of defense reactions against the oomycete Pythium aphaniderma- tum (Edson) Fitzp., including the induction of protective barriers (see above), activation of CHI, chitosanase, and GLU (Ghauoth et al., 1994). The effect of chitosan preparations against the fungus Phytophthora infestans (Mont.) de Bary during leaf treatment of potatoes manifested in an increase in the content of polyphenols in plant tissues and suppression of the growth of the pathogen (Zheng et al., 2021). In the same species, a similar effect was also demonstrated against the causative agent of early late blight Alternaria solani Sorauer (Abd El-Kareem, Haggag, 2014). In rice, several mechanisms of inhibition of bacterial pathogens have been identified by treating plant leaves with chitosan. On the one hand, there is a direct effect causing lysis of cell membranes and destruction

of bacterial biofilms, and on the other hand, an increase in the production of plant defense proteins, including oxidative stress proteins (peroxidases and oxidases), PAL, etc. (Li et al., 2013a; Stanley-Raja et al., 2021). All these mechanisms provided rice resistance to such pathogenic bacteria as Xanthomonas oryzae pv. oryzae and Xanthomonas oryzae pv. oryzicola, pathogens of bacterial late blight and leaf streak, respectively. The positive effect of leaf treatment on resistance has also been shown in other plant species (Reglinski et al., 2010; Lou et al., 2011; Li et al., 2013b).

Fruit treatment

The treatment of fruits with biostimulants is of great interest in connection with the problem of tolerance of many pathogens that develop on fruits after harvest to conventional chemical pesticides, as well as in connection with the toxicity of the latter to humans. It has been shown that chitosan reduces the rate of respiration, the production of ethylene, the aging hormone, and moisture loss, thereby contributing to the longterm preservation of the quality of fruits and vegetables (Li, Yu, 2001). Thus, the production of macerating enzymes of cell walls that destroy pectins and cellulose in sweet pepper fruits under the action of chitosan is reduced (Ghaouth et al., 1997). In cherry tomato fruits, chitosan and its complex with methyl jasmonate enhance the activity of PPO, POD, and PAL in the presence of the fungus Alternaria alternata (Fr.) Keissl. (Chen et al., 2014). Papaya fruits treated only with chitosan or chitosan in combination with plant extracts remain resistant to the anthracnose pathogen (Bautista-Baños et al., 2003). Treatment of lychee fruits with kadosan (a new formulation of chitosan) effectively reduces their sensitivity to late blight by increasing the activity of CHI, GLU, APX, as well as the accumulation of lignin during storage (Jiang et al., 2018). Chitosan treatment suppresses B. cinerea and Penicillium expansum Link fungi (causative agents of gray and blue mold, respectively) during storage of tomato fruits, through a direct fungicidal mechanism, including destruction of the spore coat, and also due to the high activity of PPO and POD in fruit tissues (Liu et al., 2007).

Another study showed that the combination of chitosan with beeswax and lime essential oil had a fungicidal effect on Rhizopus stolonifer (Ehrenb.) Vuill. by inhibiting mycelium growth, spore germination and sporulation of this fungus in potato (Ramos-García et al., 2012). W. Qing et al. evaluated the effect of chitosan on the control of Sclerotinia sclerotiorum (Lib.) de Bary (sclerotinia rot) in carrot (2015). As a result, various antipathogenic effects have been established, including damage to plasma membranes, lipid peroxidation, protein loss, along with an increase in PPO and POD activity in fruits tissues. Other authors showed that soaking harvested sweet cherries or irrigating them with a chitosan solution before harvest effectively suppresses a range of fungal pathogens, namely: B. cinerea, P. expansum, R. stolonifera, A. alternata, and Cladosporium spp. (Romanazzi et al., 2003). The reduction in infection symptoms correlated with a protective response associated with PAL accumulation. Z. Ma et al. found that chitosan-induced induction of GLU, POD, CAT, CHI, and other enzymes controls brown rot (Monillinia fructicola) affecting peach fruits (2013). However, the effect of chitosan per se was not effective in all cases. For example, it did not provide complete protection of pear fruits against blue mold (P. expansum), although it was very effective in combination with Cryptococcus laurentii and calcium chloride (Meng et al., 2010).

Plant protection products based on chitosan

Despite the presence of a large number of positive effects of chitosan in terms of plant disease control, at present, its use in its pure form is rather limited due to insufficient efficiency. An increase in the biological efficiency of preparations based on chitosan is achieved by its chemical modification, which affects the physical properties, by selecting the optimal ratio of low- and high-molecular forms of chitosan for a particular pathogen-host system, and also by creating complexes with other biologically active substances. The latter, in particular, include organic acids: salicylic, arachidonic, succinic, glutamic, etc., which induce the mechanisms of local and systemic plant resistance to pathogens and thereby increase plant productivity under adverse conditions

At the moment, a number of complex preparations have been developed in Russia, such as “Narcissus”, “Chitozar”, “Ecogel”, etc. Of particular interest is “Narcissus” (JSC Agroprom – MDT Group of Companies), which includes chitosan (50 %), succinic (30 %) and glutamic (20 %) acids. It increases the resistance of wheat to leaf rust and root rot, rice to blast, tomatoes to late blight and fusarium, cucumbers to powdery mildew, etc. (Badanova et al., 2016). In addition, the preparation destroys the chitinous membrane of rootknot nematodes (Dobrokhotov, 2000; Gol’din, 2014). “Ecogel” (Biochemical Technologies Ltd., Moscow) was obtained by magnetic enrichment of chitosan lactate with silver ions (http://ekogel.ru/poleznaya-informaciya/laktat-hitozana-dlyarasteniy- svoystva-primenenie/). It improves plant growth and root formation, increases the resistance of a number of crops, such as sugar beet, sunflower, potato, etc., to fungal, bacterial and viral diseases when applied by seed treatment and spraying of plants (Tyuterev, 2015). The All-Russian Institute of Plant Protection (St. Petersburg, Pushkin) has developed a number of preparations under the general name “Chitozar” based on chitosan and other biologically active substances. In addition to chitosan, their composition includes: SA and potassium phosphate (“Chitozar M”), arachidonic acid (“Chitozar F”). These combined preparations were effective against such pests as powdery mildew and downy mildew fungi, California thrips (Kirillova, 2015; Badanova et al., 2016). In particular, the activity of preparations with arachidonic acid and SA against Phytophthora infestans (Mont.) de Bary and virus Y, respectively, was demonstrated on potato. In the case of phytophthora, the biological efficiency of the complex was 15 % higher compared to treatment with chitosan alone, and in the case of virus Y plants showed complete resistance after treatment with the complex (6.7 % infected in plants pretreated with chitosan only) (Tyuterev, 2015).

As known, according to the type of nutrition, pathogens are classified into biotrophs, necrotrophs, and hemibiotrophs having different sensitivity to ROS, the level of which is controlled by the antioxidant system. The effect of immunomodulators based on chitosan, vanillin, and SA on the resistance of wheat to pathogens of leaf rust and dark brown spotting differing in the type of nutrition was studied. Combined preparations of chitosan with a certain ratio of vanillin and SA were developed, which provided a high antipathogenic effect against both pathogens due to the modulation of the activity of enzymes of the antioxidant system (Popova et al., 2018).

A perspective direction in plant protection is the use of a complex of chitosan with alginate – a polysaccharide that is part of the cell wall of brown algae. This complex provides encapsulation of beneficial microorganisms that can be used as probiotics and pathogen antagonists (Saberi Riseh et al., 2021).

As mentioned above, there are conflicting data on the antipathogenic activity of low and high molecular weight chitosan, which is largely due to the lack of a unified and reliable method for determining its molecular weight, as well as the fact that in most cases chitosan preparations are a mixture of molecules of different sizes. Along with the complexity and high cost of analyzing the composition of these preparations (the level of polymerization of molecules, the degree of their acetylation, etc.), some chemical features of chitosan also limit its use. For instance, the solubility and, consequently, the efficiency of chitosan in neutral or alkaline media (soil or aqueous solution) is significantly inferior to those in an acidic environment (Katiyar et al., 2014). The solubility of chitosan in a wide pH range can be increased by chemical modification of the polymer molecule, for example, by interaction with mannose (Yu et al., 2023), addition of methyl groups (Wang et al., 2015), and also by intramolecular crosslinking. Recently, a new chitosan derivative, novochizol, was obtained by the last method. Unlike the linear chitosan molecule, the novochizol molecule has a globular, close to spherical shape (https://www. novochizol.ch). Such a molecular design gives it a number of advantages over chitosan, namely: higher chemical stability, low degree of biodegradation, solubility in aqueous solutions with pH > 6, increased adhesion, and the ability to retain various active substances, such as fungicides, in globules and slowly release them. The latter feature provides a significant decrease in the effective concentrations of active substances and, accordingly, a decrease in their negative impact on ecosystems and humans.

The unique capabilities of novochizol allow to combine it with almost any substances (of low or high molecular weight, hydrophilic, hydrophobic, even insoluble), as well as bacteria, fungi and their spores, viruses. Various combination methods (by impregnation or emulsification) make it possible to control the dose of active component and its release rate, the degree of adhesion, and other parameters. It has recently been shown that treatment with novochizol stimulates the germination of common wheat seeds in the soil, and also increases both the root biomass and the total seedling biomass (by 1.5 and 1.8 times, respectively) (Teplyakova et al., 2022). Unlike chitosan, the effect of novochizol and its complexes on plant resistance to pathogens is still poorly studied. It is only assumed that such an action may have a much more pronounced effect due to the synergistic action of novochizol per se, and the action of other biologically active substances, for which it can serve as a carrier. There are already preliminary data confirming this assumption obtained on various plant objects (https://www. novochizol.ch/agrotechnology/).

## Conclusion

Among the approaches aimed at increasing the resistance of plants to certain factors, biological protection products have great prospects, since, unlike most of the chemical pesticides used, they do not pollute the environment and are non-toxic to humans. These products include chitosan, a deacetylated derivative of chitin. According to numerous authors, treatment with chitosan leads to an increase in plant biomass and an increase in their resistance to abiotic and biotic environmental factors. The antipathogenic effects of chitosan are associated both with a direct effect on pathogens and with its elicitor action associated with the induction of PAMP. The specific biological effects of chitosan are determined by the types of pathogen and host plant, environmental conditions and method of application, depending on the plant organ being treated. Despite the facts of the successful use of chitosan in agrobiology, some of its physical and chemical properties: low solubility and adhesion, chemical instability, limit this application. Recently, a number of different preparations of chitosan have been developed in combination with biologically active substances that enhance its action, as well as an improved chemical derivative, novochizol, which has great potential for use as a biostimulant and an effective plant protection agent

## Conflict of interest

The authors declare no conflict of interest.
